# Alkaloids Profiling of* Fumaria capreolata* by Analytical Platforms Based on the Hyphenation of Gas Chromatography and Liquid Chromatography with Quadrupole-Time-of-Flight Mass Spectrometry

**DOI:** 10.1155/2017/5178729

**Published:** 2017-11-28

**Authors:** María del Mar Contreras, Noureddine Bribi, Ana María Gómez-Caravaca, Julio Gálvez, Antonio Segura-Carretero

**Affiliations:** ^1^Research and Development Functional Food Centre (CIDAF), Bioregión Building, Health Science Technological Park, Avenida del Conocimiento s/n, 18016 Granada, Spain; ^2^Department of Analytical Chemistry, Faculty of Sciences, University of Granada, Avda. Fuentenueva s/n, 18071 Granada, Spain; ^3^CIBER-EHD, Department of Pharmacology, ibs.GRANADA, Center for Biomedical Research (CIBM), University of Granada, Avenida del Conocimiento s/n, Armilla, 18016 Granada, Spain; ^4^Laboratoire de Biotechnologies Végétales et Ethnobotanique, Faculté des Sciences de la Nature et de la Vie, Université de Bejaia, 06000 Bejaia, Algeria

## Abstract

Two analytical platforms, gas chromatography (GC) coupled to quadrupole-time-of-flight (QTOF) mass spectrometry (MS) and reversed-phase ultrahigh performance liquid chromatography (UHPLC) coupled to diode array (DAD) and QTOF detection, were applied in order to study the alkaloid profile of* Fumaria capreolata*. The use of these mass analyzers enabled tentatively identifying the alkaloids by matching their accurate mass signals and suggested molecular formulae with those previously reported in libraries and databases. Moreover, the proposed structures were corroborated by studying their fragmentation pattern obtained by both platforms. In this way, 8 and 26 isoquinoline alkaloids were characterized using GC-QTOF-MS and RP-UHPLC-DAD-QTOF-MS, respectively, and they belonged to the following subclasses: protoberberine, protopine, aporphine, benzophenanthridine, spirobenzylisoquinoline, morphinandienone, and benzylisoquinoline. Moreover, the latter analytical method was selected to determine at 280 nm the concentration of protopine (9.6 ± 0.7 mg/g), a potential active compound of the extract. In conclusion, although GC-MS has been commonly used for the analysis of this type of phytochemicals, RP-UHPLC-DAD-QTOF-MS provided essential complementary information. This analytical method can be applied for the quality control of phytopharmaceuticals containing* Fumaria* extracts currently found in the market.

## 1. Introduction

In the “Conspiracy of Cortes” the Spanish novelist Matilde Asensi mentions the usage of* Fumaria* as a natural treatment for bubonic plague [1]. This fact is not far from fiction since the genus* Fumaria* (Fumarioideae, Papaveraceae) has traditionally been used in traditional medicine [2]. This genus consists of above 46 species widespread in the world, which grows in wheat fields, plains, and low hills in Europe, Middle East, South Asia, and so on [3]. The biological activities of* Fumaria* spp. are linked to the presence of key compounds such as isoquinoline alkaloids [4]. In fact, alkaloids, including the aforementioned type, have recently been reviewed to be effective treatments of intestinal inflammation injury in animal models [5]. Interestingly, recent studies have shown the low toxicity of a* Fumaria capreolata* alkaloid extract (white ramping-fumatory)* in vitro* and* in vivo *[2], as well as their intestinal anti-inflammatory effects in mice colitis [6] and antinociceptive activity [7].

Isoquinoline alkaloids derive from the amino acid tyrosine, after its conversion to 3,4-dihydroxyphenylethylamine (dopamine) and 4-hydroxyphenylacetaldehyde that act as intermediary molecules [8]. This type of alkaloids has been characterized by thin layer chromatography [9], gas chromatography (GC) coupled to flame ionization detector (FID) [10], and mass spectrometry (MS) [11–14], as well as liquid chromatography (LC) coupled to UV/Vis, diode array detection (DAD) [15], and mass spectrometry (MS) [16]. In the case of GC-MS, there is available standard mass spectral data library for the characterization of several phytochemical classes, but it is not at all complete for alkaloids in comparison with other phytochemicals and explained by their relatively poor volatilization [17]. In the case of LC-MS, there are efforts to generate spectral libraries using different MS analyzers and collision energies due to the little lack of consistency, standardization, or reproducibility as compared with GC-MS or nuclear magnetic resonance spectroscopy [18]. MassBank (http://www.massbank.jp/) and Metlin (https://metlin.scripps.edu/) are examples of public repositories of tandem mass spectral data.

Recent studies have demonstrated the potential of the hybrid mass analyzer quadrupole-time-of-flight (QTOF) for the profiling of hundreds of natural plant metabolites. This type of mass analyzers provides excellent selectivity and mass accuracy over a wide dynamic range and elucidates the molecular formula of unknown compounds. It also performs tandem MS, which is useful for a preliminary structural elucidation [19–21] when information in databases is limited. Thus, the objective of this study was to explore the potential of GC and LC coupled to QTOF mass analyzer for the profiling of isoquinoline alkaloids in* F. capreolata*. This plant was selected due to its aforesaid biological properties. In addition, to the best of our knowledge, there are not any reports in the literature accomplishing the comparison of these two analytical platforms for the qualitative characterization of this type of phytochemicals.

## 2. Experimental

### 2.1. Preparation of the Alkaloid Extract

Aerial parts of* F. capreolata* were collected from Bejaia area (Algeria) in May 2012, when they were at the flowering and fruit setting stage. Plants were authenticated by Dr. F. Maiza-Benabdesselam (Laboratory of Plant Biotechnology and Ethnobotany, University of Bejaia, Algeria) and voucher specimen was deposited (Reference number FC015). Aerial parts of the plants were dried in oven at 40°C overnight and ground. In brief, the powder samples were extracted as described before [2, 7] with methanol and dichloromethane to afford a crude extract of alkaloids. For analysis, the alkaloid extract (10 mg) was dissolved in methanol (1 mL) (ThermoFisher, Waltham, MA, USA) and filtered with a 0.20 *μ*m syringe filter of polytetrafluoroethylene (13 mm) (ThermoFisher).

### 2.2. Analysis by GC-QTOF-MS

Analyses were carried out using an Agilent technologies (Palo Alto, CA, USA) gas chromatography (GC) system 7890B (G3440B) coupled to a 7200 accurate mass quadrupole-time-of-flight (Q-TOF) mass spectrometer. A capillary column was employed: HP-5MS (5% phenyl 95% dimethylpolysiloxane, 30 m × 0.250 mm i.d.; 0.25 *μ*m film thickness). The column temperature was initially held at 200°C for 1 min, increased at 25°C/min to 250°C, and then held at 250°C for 31 min. Finally, a ramp of 10°C/min was applied until 310°C and maintained for 5 min. The total run was 45 min. The helium flow rate was 1.2 mL/min. The injection program included sequential washing steps of the syringe and a sample pumping step for removal of small air bubbles. The injection was 1 *μ*L using a programmable PAL GC sampler 120 and with a split ratio of 10 : 1. The inlet and the transfer line temperatures were 250°C and 300°C, respectively. The ion source was electron impact (EI) carried out at 70 eV and the temperature was 250°C. The mass range from* m/z* 125 to 700 was scanned at a rate of 3 scans/s. The TOF analyzer was calibrated previously to each analysis using an Agilent calibration tune mix.

### 2.3. Analysis by Reversed-Phase Ultrahigh Performance Liquid Chromatography Coupled to Diode Array Detection (DAD) and QTOF-MS

Analyses were made with an Agilent 1200 series rapid resolution (Palo Alto, CA, USA) equipped with a binary pump, an autosampler, and a DAD. The mobile phases consisted of a water with 0.2% formic acid (mobile phase A) and acetonitrile (mobile phase B), and a multistep linear gradient was applied: 0–5.5 min, 1–7% B; 5.5–11 min, 7–14% B; 11–17.5 min, 14–24% B; 17.5–22.5 min, 24–40% B; 22.5–27.5 min, 40–100% B; 27.5–28.5 min, 100-100% B; 28.5–29.5 min, 100-1% B. The latter value (99% A and 1% B) was held for 5.5 min to equilibrate the column with the initial conditions prior to the next injection. The total run was 35 min. The flow rate was set at 0.5 mL/min throughout the gradient. Separation was carried out with a Zorbax Eclipse XDB-C18 column (4.6 × 50 mm, 1.8 *μ*m of particle size) (Agilent) at 25°C. The UV-Vis spectra were recorded from 190 to 600 nm, and a wavelength channel of 280 ± 4 nm was applied for quantitative purposes. The injection volume was 1 *μ*L.

The spectra were acquired in negative and positive ion modes over a mass-to-charge (*m/z*) range from 70 to 1500. The operation conditions were set in the adequate polarity as follows: gas temperature: 325°C; drying gas: nitrogen at 10 L/min; nebulizer pressure: 20 psig; sheath gas temperature: 400°C; sheath gas flow: nitrogen at 12 L/min; capillary voltage: 4000 V; skimmer: 45 V; octapole radiofrequency voltage: 750 V; focusing voltage: 500 V, with the corresponding polarity automatically set. The acquisition mode was AutoMS2.

Internal mass correction of each sample was performed with a continuous infusion of Agilent TOF mixture containing two mass references for each ionization mode. In the positive ionization mode, the two reference mass ions were at* m/z* 121.0509 (purine) and 922.0098 (hexakis (1H, 1H, 3H-tetrafluoropropoxy) phosphazine). Alternatively, in the negative ionization mode trifluoroacetic acid ammonium salt (*m/z* 112.9856 that is trifluoroacetic acid) and hexakis (1H, 1H, 3H-tetrafluoropropoxy) phosphazine (*m/z* 1033.9881 corresponding to the trifluoroacetic acid ammonium salt adduct) were used. The detection window was set to 100 ppm. Data acquisition (2.5 Hz) in the profile mode was governed* via* the Agilent MassHunter Workstation B.05.01.

Data analysis was performed on a Mass Hunter Qualitative Analysis B.06.00, as commented before. The isotope model selected was “common organic molecules” with a peak spacing tolerance of* m/z* 0.0025 and 7 ppm.

All analyses by GC-QTOF-MS and RP-HPLC-DAD-QTOF-MS were done in triplicate.

### 2.4. Data Processing

Data analysis was performed on MassHunter Qualitative Analysis B.06.00 (Agilent technologies). The characterization of compounds was performed by generation of candidate formula with a mass accuracy limit of 5 ppm for the analysis of RP-UHPLC-DAD-QTOF-MS and 10 ppm for GC-QTOF-MS, and also considering the MS score that should be close to 100. The latter parameter is related to the contribution to mass accuracy, isotope abundance, and isotope spacing for the generated molecular formulae. Using GC-QTOF-MS, alkaloids were characterized by direct comparison of their fragmentation pattern with data from the NIST MS library (NIST11.L) and NIST chemistry WebBook (http://webbook.nist.gov/chemistry/) when possible, as well as literature about Papaveraceae. In the case of RP-UHPLC-DAD-QTOF-MS and –MS/MS, MassBank and Metlin were consulted. Moreover, chemical structure information was also retrieved from SciFinder Scholar (https://scifinder.cas.org), Reaxys (http://www.reaxys.com), and KNApSAcK Core System (http://kanaya.naist.jp/knapsack_jsp/top.html).

### 2.5. Quality Control of Protopine

Protopine hydrochloride stock solution was conveniently diluted with methanol to prepare calibration points (8–260 nmol/mL). The external standard method was used and a linear regression for the calibration curve was estimated using the area under the curve of protopine against concentration. Repeatability was assayed by three consecutive injections of the methanolic solutions of at three levels three times (intraday repeatability) and five times on two different days (interday repeatability) [22]. The limit of detection (LOD) and quantification (LOQ) were estimated as protopine concentration giving a signal equal to the blank signal plus three and ten standard deviations of the blanks, respectively [23].

## 3. Results and Discussion

### 3.1. Alkaloidal Profiling* via* GC-TOF-MS

**Figure 1:**
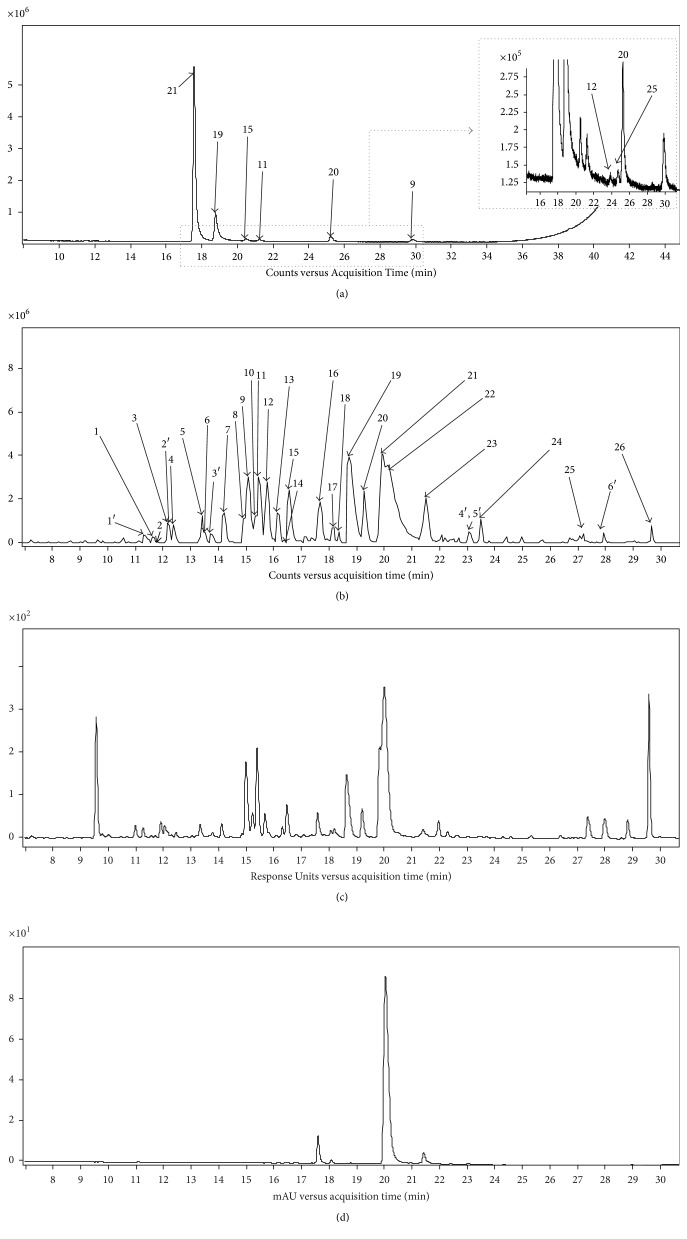
(a) Total ion chromatogram obtained by GC-QTOF-MS, (b) base peak chromatogram in the positive ionization mode, (c) chromatograms at 280 nm, and (d) 450 nm of the alkaloid extract obtained by RP-UHPLC–DAD–QTOF-MS. Compounds are numbered according to Table 2: (1) pallidine; (2) isoboldine derivative; (3)* N,N*-dimethylcoclaurine (4) fumaritine; (5) coclaurine; (6)* N*-methylcoclaurine; (7) magnoflorine; (8) reticuline; (9) parfumine; (10) parfumidine; (11) isoboldine; (12) coreximine; (13) methylcoreximine 1; (14) methylcoreximine 2; (15) cheilanthifoline; (16) dehydrocheilanthifoline; (17) demethyleneberberine/jatrorubine; (18) cryptopine; (19) protopine; (20) fumariline; (21) stylopine; (22) coptisine; (23) corysamine; (24) impatien C; (25) dihydrosanguinarine; (26) 8-oxocoptisine. 1′–6′ unknown compounds.

Briefly, for the untargeted analysis of the alkaloidal extract* via* GC-TOF-MS the strategy followed consisted of the generation of the molecular formulae of the detected ions in the chromatographic profile (Figure 1(a)) and studying the fragmentation pattern using NIST MS library when possible or based on literature [11, 12, 14, 24]. The results provided by the GC–TOF-MS analyzer are given in Table 1, which shows MS experimental data, retention time (RT), and main fragments generated by EI from* F. capreolata* alkaloids. Table S1 (in Supplementary Material available online at https://doi.org/10.1155/2017/5178729) additionally provides literature about their occurrence in* F. capreolata*, fragmentation pattern in GC-MS, and information found in databases. Using this approach, a total of eight isoquinoline alkaloids were characterized and eluted in the following order: stylopine, protopine, cheilanthifoline, isoboldine, coreximine, dihydrosanguinarine, fumariline, and parfumine. These alkaloids were representatives of protoberberine, protopine, benzophenanthridine, aporphine, and spirobenzylisoquinoline types. These results agreed with previous studies on this plant using GC coupled to a quadrupole mass analyzer [11, 12] and other studies [9, 25], while additionally we also detect coreximine. Moreover, in agreement with previous studies [13, 14, 17, 24] our results suggest that the stationary phase 5% phenyl 95% dimethyl polysiloxane is also an adequate alternative to the more apolar stationary phases consisting of 100% dimethyl polysiloxane, which is also commonly used for the separation of isoquinoline alkaloids [11, 12].

EI source is considered a hard ionization method since it provokes extensive fragmentation by high energetic electrons. Interestingly, the TOF mass analyzer enabled us to detect the molecule ions (M^+^) of each alkaloid in itself and generate their molecular formulae with errors lower than 10 ppm (Table 1 and Table S1). It seems that this platform is highly attractive since it can provide not only the fragmentation pattern of alkaloids, but also their molecular formulae, a basic clue for structure elucidation of unknown compounds that are not available in current GC-MS libraries. This was the case of dihydrosanguinarine and parfumine (Table S1), whose structures were proposed based on both their molecular formula and the fragmentation pattern that could be fortunately compared with literature [11, 14, 24].

**Figure 2:**
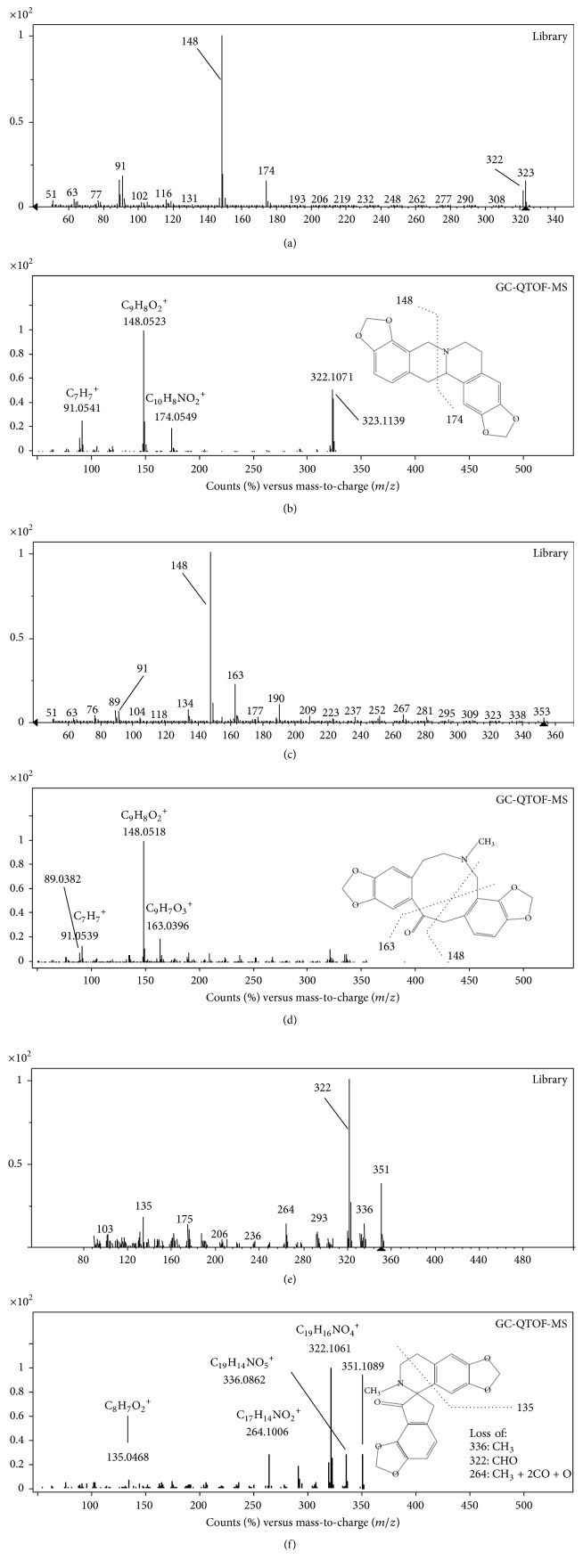
Fragmentation patterns of stylopine ((a) and (b)), protopine ((c) and (d)), and fumariline ((e) and (f)) described in the library and determined by GC-QTOF-MS, respectively.

As an example of the strategy followed, Figure 2 shows the fragmentation pattern of stylopine, protopine, and fumariline using GC-QTOF-MS.

### 3.2. Alkaloidal Profiling* via* RP-HPLC-DAD-QTOF-MS

Preliminary* F. capreolata *alkaloids were studied using ESI in both negative and positive ionization modes. The second ionization mode led to a richer and complex chromatographic profile (Figure S1) with more intense signals. Thus, it was selected for further studies. This is not surprising since most LC-MS methods have applied the latter ionization mode for the analysis of alkaloids. As an example, the alkaloid profile of* F. capreolata* is shown in Figure 1(b). It depicts the base peak chromatogram (BPC) under the selected analytical conditions, as well as the chromatograms at 280 nm, at which isoquinoline alkaloids show absorption, and 450 nm, which is particular of quaternary protoberberine alkaloids.

**Figure 3:**
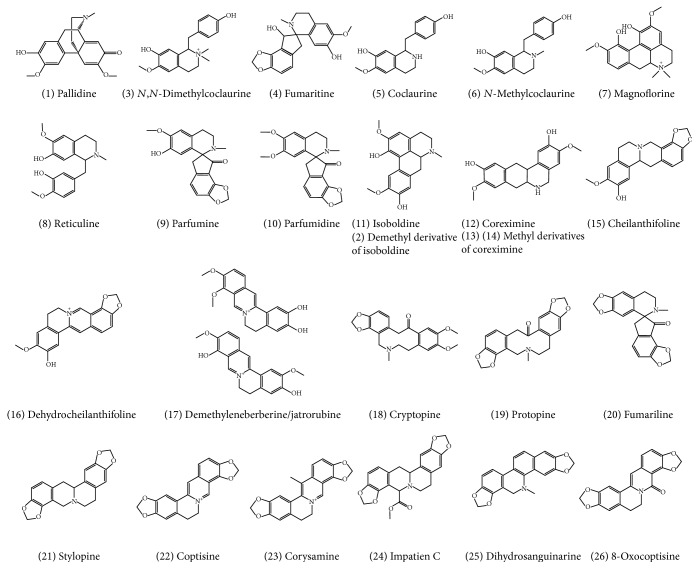
Chemical structures of the alkaloids present in* F. capreolata* extract and characterized by the QTOF platforms. Compounds are numbered according to Table 2.

Afterwards, using this analytical platform, the characterization of the alkaloids was based on the following strategy described in our previous studies on other plant phytochemicals [20]. Firstly, the UV-Vis spectra and the generation of the molecular formulae enabled proposing the chemical structures of the alkaloids. For that, literature on* Fumaria* spp. [9, 11, 12, 25, 26] and the aforementioned chemical databases were consulted. Secondly, the MS/MS spectra were studied in depth. In this way, 26 alkaloids were characterized on the basis of their spectrometric data and the results are shown in Table 2 and Table S2: RT, molecular formula, observed* m/z*, mass error, MS score, UV-Vis maximums, and main MS/MS fragments. As before, Table S2 includes additional information. The chemical structures are depicted in Figure 3.

**Figure 4:**
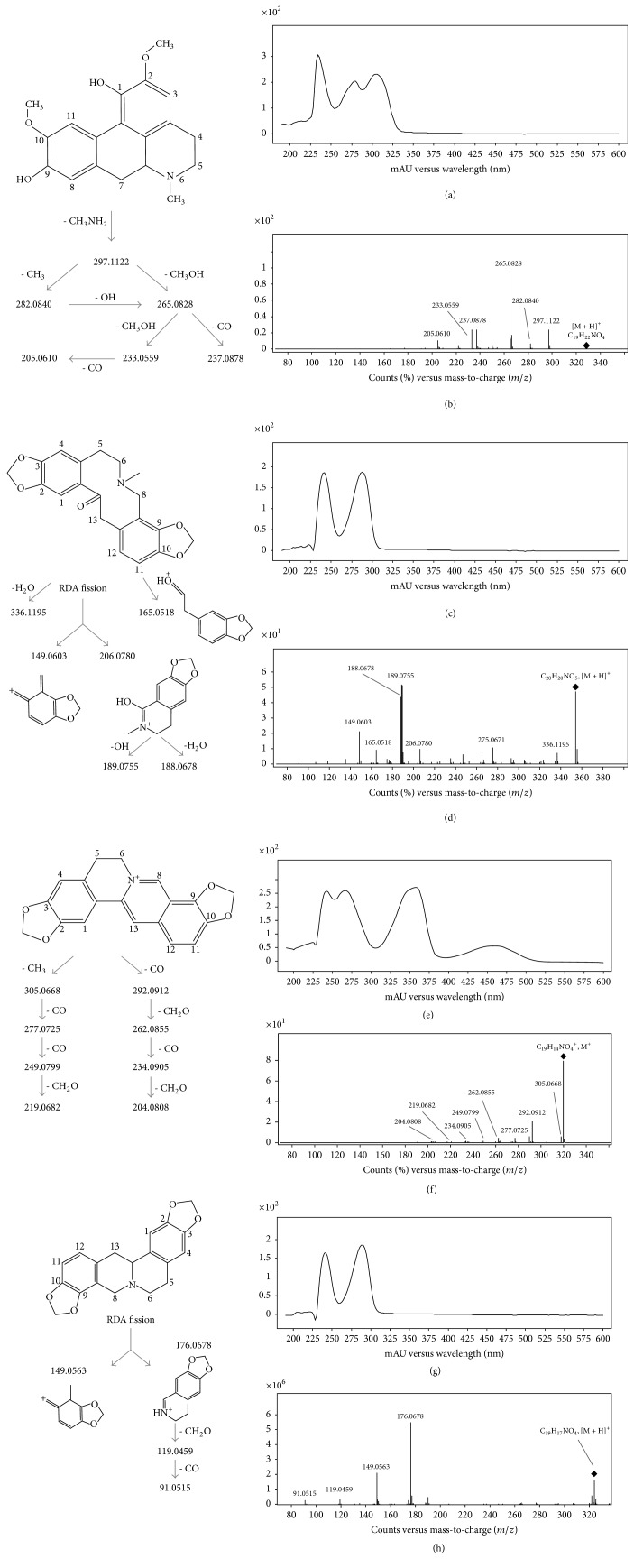
UV-Vis spectra and fragmentation pattern of isoboldine ((a) and (b)), protopine ((c) and (d)), coptisine ((e) and (f)), and stylopine ((g) and (h)), respectively, determined by RP-UHPLC-DAD-QTOF-MS.

Using the positive ionization mode, the tertiary alkaloids led [M+H]^+^ ions, whereas quaternary alkaloids yielded [M]^+^ ions in the mass spectra that is in accordance with Ding et al. [27]. Moreover, the different types of alkaloids showed different UV absorption spectra with maximum absorption between 260 and 290 nm (Table 2 and Table S2). Interestingly, UV-Vis spectroscopy is particularly useful for the elucidation of quaternary isoquinoline alkaloids. For example, UV absorption spectra of quaternary protoberberine alkaloids, 16, 17, 22, and 23, are determined by the auxochromic groups bound to ring D with a minimum at 301–310 nm, indicating a protoberberine core with substituents on carbons C9 and C10 [28] and also presented a characteristic maximum absorption around 450 nm, in agreement with Grycováa et al. [29]. As an example, see coptisine in Figures 3 and 4.

Once the molecular formulae were generated and the UV-Vis results contrasted, the MS/MS fragmentation patterns were studied and compared with those in databases, but only experimental MS/MS spectra of protopine and cryptopine were found (Table S2). However, most of the MS/MS spectra shared common ions with those found in studies on plants containing alkaloids from Papaveraceae, Lauraceae, and Rutaceae [27, 30–35] being useful to define the chemical structure of the alkaloids. As an example, Figure 4 shows the fragmentation pattern of isoboldine, protopine, coptisine, and stylopine (tetrahydrocoptisine), as examples of aporphine, protopine, and both protoberberine quaternary and tertiary alkaloids, respectively. The fragmentation patterns of isoboldine and coptisine were characterized by a cleavage of the substituted groups of the alkaloid core, whereas no ring fusion was observed. In this sense, isoboldine (*m/z* 328) shows the primary loss of CH_3_NH_2_ (31 Da) at* m/z* 297 due to the presence of a methyl substituent in the amino group, as it was observed for its derivative (*m/z* 314) at* m/z* 283. This is a characteristic of this type of aporphine alkaloids [36]. Afterwards, the loss of CH_3_, CH_3_, OH, CH_3_OH, and CO occurred. In the case of protopine, their product ions were generated by dehydration (*m/z* 336), retro-Diels-Alder fragmentation (*m/z* 149 and 206), and subsequent losses of H_2_O (*m/z* 188) and OH (*m/z* 189), in accordance with Shim et al. [37] and Schmidt et al. [30]. The fragmentation of the protopine backbone was also observed by GC-QTOF-MS as it was shown in Figure 2. Similarly, the fragmentation of the backbone of stylopine generated fragment ions at* m/z* 176 and 149, which were the most abundant. These ion fragments were also observed in the MS/MS spectrum of the compound 24 (*m/z* 396, C_22_H_21_NO_6_) (Table 2), together with the fragment at* m/z* 322 released after the primarily neutral loss of a substituent with molecular formula C_3_H_6_O_2_ (74 Da). Therefore, this compound was tentatively identified as impatien C, a protoberberine recently characterized in* Corydalis impatiens* (Fumarioideae, Papaveraceae) [38].

Dihydrosanguinarine (benzophenanthridine type) was also characterized by the fragmentation of their substituents, being the most abundant ions formed by the loss of CH_3_ (*m/z* 319), CH_2_O (*m/z* 304), and CO (*m/z* 276) (Table 2). Benzylisoquinolines presented the loss of NH_3_ (coclaurine), CH_3_NH_2_ (*N*-methylcoclaurine), or C_2_H_6_NH (*N,N*-dimethylcoclaurine) at* m/z* 269, substituents of the isoquinoline core, and also shared a common ion at* m/z* 107 (C_7_H_7_O^+^), which correspond to the methylphenol moiety generated by inductive cleavage [37]. Finally, spirobenzylisoquinolines showed not only the neutral loss of substituents of the alkaloid core but also the breakage at the isoquinoline core generating ions at* m/z* 177 (C_10_H_11_NO_2_) (fumariline), 179 (C_10_H_13_NO_2_) (parfumidine), and 193 (C_11_H_15_NO_2_) (parfumine), and its subsequent fragmentation (e.g.,* m/z* 135 and 137).

### 3.3. Comparison of the QTOF Platforms

**Figure 5:**
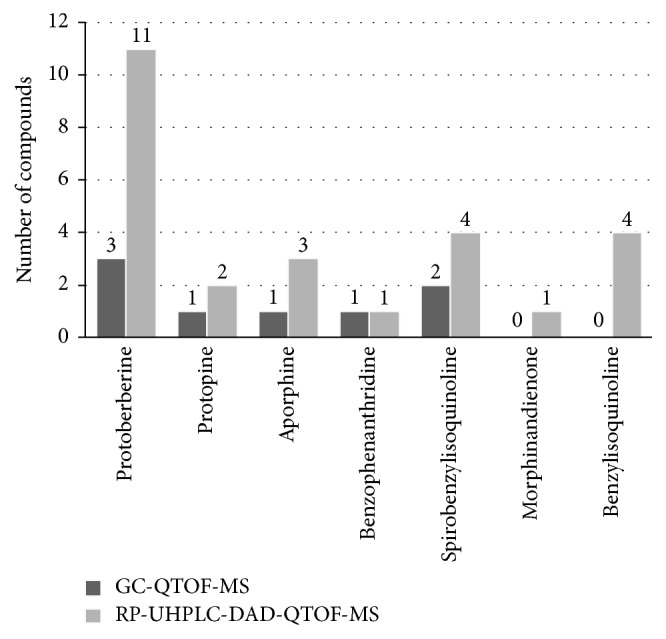
Comparison summary of the qualitative analysis of* F. capreolata *alkaloids by GC-QTOF-MS and RP-UHPLC-DAD-QTOF-MS.

Figure 5 represents a comparative plot of the results described above. All the isoquinoline alkaloids found by GC-TOF-MS in the extract of* F. capreolata* were also detected using RP-HPLC-DAD-QTOF-MS. More polar alkaloids, such as quaternary alkaloids, were not detected by the first analytical platform probably due to their poor volatility that is its major drawback [17]. Moreover, the use of RP-HPLC-DAD-QTOF-MS enables us to find some alkaloids, which were not reported in this plant by other authors (e.g., compounds 2, 3, 5, 10, 13, 14, 17, 23, 24, and 26). This can be explained by the fact that most of the studies on* Fumaria* spp. used GC-MS for studying their alkaloids profile.

A recent study has shown the trends of using ESI, EI, and matrix-assisted laser desorption/ionization (MALDI) as ionization sources for natural products investigations; the application of ESI source is continuously growing, while MALDI and EI remain constant [39]. This trend possibly reflects the huge expansion of ESI applications in this area explained by the fact that MS is a versatile detection system and ESI source enables the analysis of a wide range of chemical structures, as our results revealed.

### 3.4. Quantification of Protopine

Protopine exhibits anti-inflammatory activity both* in vitro* and* in vivo* [40, 41]. This alkaloid can contribute, at least in part, to explaining the antinociceptive and anti-inflammatory effects of the* F. capreolata* extract showed in our previous studies [2, 6, 7]. Thus, for the quality control of this alkaloid extract, protopine was preliminary selected as analytical/active marker since it is also commercially available and the RP-HPLC method was selected since it was shorter than the GC method. Moreover, the quantification of this compound was performed at 280 nm. This detector was selected since it is cheap and common in pharmaceutical/plant industries, which can be interested in reproducing the extraction protocol of isoquinoline alkaloids from* F. capreolata *or for further standardization purposes. The regression equation was* y* = 1.78*x* + 7.43. A *R*^2^ of 0.999 was obtained, indicating a good correlation. The repeatability met quality criteria, with relative standard deviation values lower than 10% and accuracy values close to 100%, respectively [42] (Table S3). The LOD and LOQ were 0.2 and 6.6 nmol/mL. Finally, using this method the estimated amount of protopine was 9.6 ± 0.7 mg/g.

The proposed analytical method can also be expanded for the quality control of phytopharmaceuticals containing* Fumaria* extracts, which can currently be found in the market without a detailed description of their active constituents.

## 4. Conclusions

GC and RP-UHPLC coupled to a high resolution QTOF mass analyzer are powerful analytical platforms for a quick structure determination of isoquinoline alkaloids, previously to the application of other spectroscopic tools. Although GC-QTOF-MS provided structural information about 8 alkaloids, RP-HPLC-DAD-QTOF-MS gave a more exhaustive profiling of* F. capreolata* alkaloids (26 characterized compounds). The latter analytical method seems to be a requirement to detect quaternary alkaloids based on our results and previous literature about this plant. Moreover, novel alkaloids were characterized in this plant when using both methods, but this number was higher when using RP-UHPLC-DAD-QTOF-MS, such as a demethylated derivative of isoboldine, coclaurine,* N,N*-dimethylcoclaurine, and 8-oxocoptisine, among others.

## Supplementary Material

Figure S1: Base peak chromatogram of *F. capreolata* extract determined by RP-UHPLC-DAD-QTOF-MS and elecrospray ionization in the negative and positive ionization modes.
Table S1: Characterization of alkaloids from *F. capreolata* extract by GC-QTOF-MS.
Table S2: Characterization of alkaloids from *F. capreolata* extract by RP-UHPLC-QTOF-MS.
Table S3: Precision and accuracy of the method proposed.

## Figures and Tables

**Table 1:** Alkaloids characterized in *F. capreolata *by GC-QTOF-MS.

Peak N°	RT^a^ (min)	Proposed alkaloid	Precursor *m/z* (M^+^)	Molecular formula	MS score	Mass error (ppm)	EI-MS *m/z* (relative intensity, %)	Alkaloid type
1	17.5	Stylopine	323.1139	C_19_H_17_NO_4_	94	3.96	323.1139 (44), 322.1071 (52), 148.0523 (100), 91.0541 (26)	Protoberberine

2	18.7	Protopine	353.1247	C_20_H_19_NO_5_	94	3.53	163.0396 (19), 149.0564 (11), 148.0518 (100), 91.0539 (14)	Protopine

3	20.4	Cheilanthifoline	325.1296	C_19_H_19_NO_4_	91	3.86	325.1289 (40), 324.1215 (46), 149.0581 (26), 148.0518 (100)	Protoberberine

4	21.2	Isoboldine	327.1438	C_19_H_21_NO_4_	80	8.09	327.1438 (54), 326.1375 (100), 284.1048 (32), 253.0833 (27)	Aporphine

5	23.8	Coreximine	327.1428	C_19_H_21_NO_4_	69	3.57	327.1436 (57), 326.1376 (63), 178.0841 (100), 176.069 (45)	Protoberberine

6	24.7	Dihydrosanguinarine	333.0971	C_20_H_15_NO_4_	83	7.01	334.1014 (18), 333.0971 (88), 332.0899 (100), 105.0669 (15)	Benzophenanthridine

7	25.2	Fumariline	351.1089	C_20_H_17_NO_5_	96	3.5	336.0862 (28), 323.1104 (25), 322.1061 (100), 264.1006 (29)	Spirobenzylisoquinoline

8	29.8	Parfumine	353.1246	C_20_H_19_NO_5_	89	4.4	353.1246 (17), 338.1006 (29), 325.1252 (26), 324.1215 (100), 322.086 (31), 308.126 (31)	Spirobenzylisoquinoline

^a^RT: retention time and EI-MS: electron impact-mass spectrometry.

**Table 2:** Alkaloids characterized in *F. capreolata *by RP-UHPLC-DAD-QTOF-MS.

Peak N°	RT^a^ (min)	Proposed alkaloid	Molecular formula	Exp.^a^ *m/z* ([M+H]^+^/M^+^)	Error (ppm)	Score	UV max._ _^a^ (nm)	Main fragments in MS/MS	Alkaloid type
1	11.59	Pallidine	C_19_H_21_NO_4_	328.1544	−0.6	98.9	282	297.1051, 265.0834, 251.0635, 243.0954, 237.0846, 211.0704, 192.0962	Morphinandienone

2	11.77	Derivative of isoboldine (demethyl)	C_18_H_19_NO_4_	314.1381	3.79	91.27	268, 306	283.0920, 268.0680, 251.0664, 233.0555, 223.0716, 205.0617	Aporphine

3	12.14	*N,N*-Dimethylcoclaurine	C_19_H_24_NO_3_^+^	314.1763	−3.87	95.1	260, 358	299.1133, 298.1061, 269.1159, 237.0903, 209.0957, 175.0762, 121.0610, 107.0497	Benzylisoquinoline

4	12.33	Fumaritine	C_20_H_21_NO_5_	356.1481	0.6	94.4	283	338.1313, 277.0788, 249.0847, 137.0541	Spirobenzylisoquinoline

5	13.32	Coclaurine	C_17_H_19_NO_3_	286.1427	2.0	88.0	286	269.1131, 237.0864, 209.0921, 175.0723, 145.0631, 143.0457, 107.0460	Benzylisoquinoline

6	13.44	*N*-Methylcoclaurine	C_18_H_21_NO_3_	300.1585	3.0	96.0	282	269.1105, 237.0846, 209.0902, 177.0489, 175.0692, 145.0232, 107.0445	Benzylisoquinoline

7	14.12	Magnoflorine	C_20_H_24_NO_4_^+^	342.1701	−0.4	99.5	268, 305	297.1081, 282.0853, 265.0828, 237.0915, 191.0832	Aporphine

8	14.80	Reticuline	C_19_H_23_NO_4_	330.1696	1.6	96.6	281	192.0990, 175.0719, 143.0460, 137.0564	Benzylisoquinoline

9	15.05	Parfumine	C_20_H_19_NO_5_	354.1341	−1.4	97.2	271, 372	336.1191, 323.0927, 305.0774, 295.0930, 279.0611, 179.0912, 137.0567	Spirobenzylisoquinoline

10	15.29	Parfumidine	C_21_H_21_NO_5_	368.1478	3.7	93.5	290	323.0859, 305.0756, 293.0754, 261.0502, 193.1053, 137.0556	Spirobenzylisoquinoline

11	15.42	Isoboldine	C_19_H_21_NO_4_	328.1546	−0.4	98.4	279, 304	297.1125, 282.0840, 265.0826, 237.0877, 233.0559, 205.0610	Aporphine

12	15.73	Coreximine	C_19_H_21_NO_4_	328.1535	2.4	96.5	282	313.1226, 178.0803, 151.0693, 119.0436, 91.0493	Protoberberine

13	16.03	Methylcoreximine 1	C_20_H_23_NO_4_	342.1701	−0.3	99.1	282	327.1431, 192.0989, 177.0754, 151.0728, 137.0573	Protoberberine

14	16.47	Methylcoreximine 2	C_20_H_23_NO_4_	342.1701	−0.4	97.3	280	192.0976, 177.0743, 151.0712	Protoberberine

15	16.53	Cheilanthifoline	C_19_H_19_NO_4_	326.1405	−1.1	90.6	285	311.1157, 192.0988, 178.0829, 151.0721, 119.0461, 91.0517	Protoberberine

16	17.58	Dehydrocheilanthifoline	C_19_H_16_NO_4_^+^	322.1074	0.3	99.3	267, 355, 458	307.0755, 294.1039, 279.0808, 264.0663, 250.0778, 222.0839	Protoberberine

17	18.11	Demethyleneberberine/jatrorubine	C_19_H_18_NO_4_^+^	324.1217	3.8	92.4	276, 355	309.1003, 294.0765, 266.0814, 210.0910	Protoberberine

18	18.32	Cryptopine	C_21_H_23_NO_5_	370.1603	1.2	97.2	283	291.1008, 222.1115, 204.1006, 190.0853, 165.0898, 165.0536, 149.0582	Protopine

19	18.69	Protopine	C_20_H_19_NO_5_	354.1342	−0.9	96.4	288	206.0780, 189.0755, 188.0678, 165.0518, 149.0603	Protopine

20	19.24	Fumariline	C_20_H_17_NO_5_	352.1187	−2.8	93.0	270, 372	334.1026, 309.0703, 279.0601, 263.0653, 177.0740, 135.0404	Spirobenzylisoquinoline

21	19.86	Stylopine	C_19_H_17_NO_4_	324.1232	−0.4	99.3	288	176.0678, 149.0563, 119.0459, 91.0515	Protoberberine

22	20.05	Coptisine	C_19_H_14_NO_4_^+^	320.0917	−1.2	90.1	265, 357, 459	305.0668, 292.0912, 277.0725, 262.0855, 249.0779, 234.0905	Protoberberine

23	21.47	Corysamine	C_20_H_16_NO_4_^+^	334.1068	3.4	95.6	268, 345, 449	320.0909, 306.1083, 291.0843, 276.0974, 261.0741, 248.1019	Protoberberine

24	23.44	Impatien C	C_22_H_21_NO_6_	396.1454	−2.9	96.3	288	322.1019, 176.0671, 149.0557, 119.0452, 91.0507	Protoberberine

25	29.62	Dihydrosanguinarine	C_20_H_15_NO_4_	334.1079	−2.8	90.7	277	319.0792, 318.0723, 317.0703, 304.0958, 276.0972, 274.0812	Benzophenanthridine

26	27.15	8-Oxocoptisine	C_19_H_13_NO_5_	336.0858/358.0703_ _^b^	9.16/−4.82_ _^b^	71.5/92.22_ _^b^	277, 330	320.0532, 308.0889, 278.0784, 280.0966, 174.0520	Protoberberine

Others									

1′	11.28	Unknown	C_11_H_22_NO_6_	265.153	−3.7	95.0	290, 318	177.0511, 145.0256, 117.0313, 89.0364	

2′	12.21	Unknown	C_19_H_17_NO_5_/C_19_H_18_NO_5_^+^	340.1188	−2.95	92.67	ND	322.1015, 309.0697, 291.0601, 279.0597, 281.0766, 263.0645, 251.0653, 233.0547, 205.0592, 165.0772, 123.0402	

3′	13.69	Unknown	C_19_H_21_NO_4_/C_19_H_22_NO_4_^+^	328.1530	4.4	91.0	ND	297.1074, 282.0847, 265.0817, 237.0868, 209.0923, 191.0823	

4′	23.01	Unknown	C_25_H_25_NO_5_	420.1836	0.43	98.51	282	364.1485, 321.0935, 292.0956	

5′	23.07	Unknown	C_26_H_19_NO_5_/C_26_H_20_NO_5_^+^	426.1341	−1.18	96	ND	398.1341, 320.0867, 107.0468	

6′	27.89	Unknown	C_24_H_17_NO_7_	432.1044	7.86	77.28	330	241.0425, 219.0608, 191.0698	

^a^Exp.: experimental; max.: maximum; RT: retention time; ^b^[M+Na]^+^.
