# Supplementary figures and images for: Alkaloids Profiling of Fumaria capreolata by Analytical Platforms Based on the Hyphenation of Gas Chromatography and Liquid Chromatography with Quadrupole-Time-of-Flight Mass Spectrometry
Source: Int J Anal Chem. 2017 Nov 28;2017:5178729. doi: 10.1155/2017/5178729 (PMC5733964; doi:10.1155/2017/5178729)

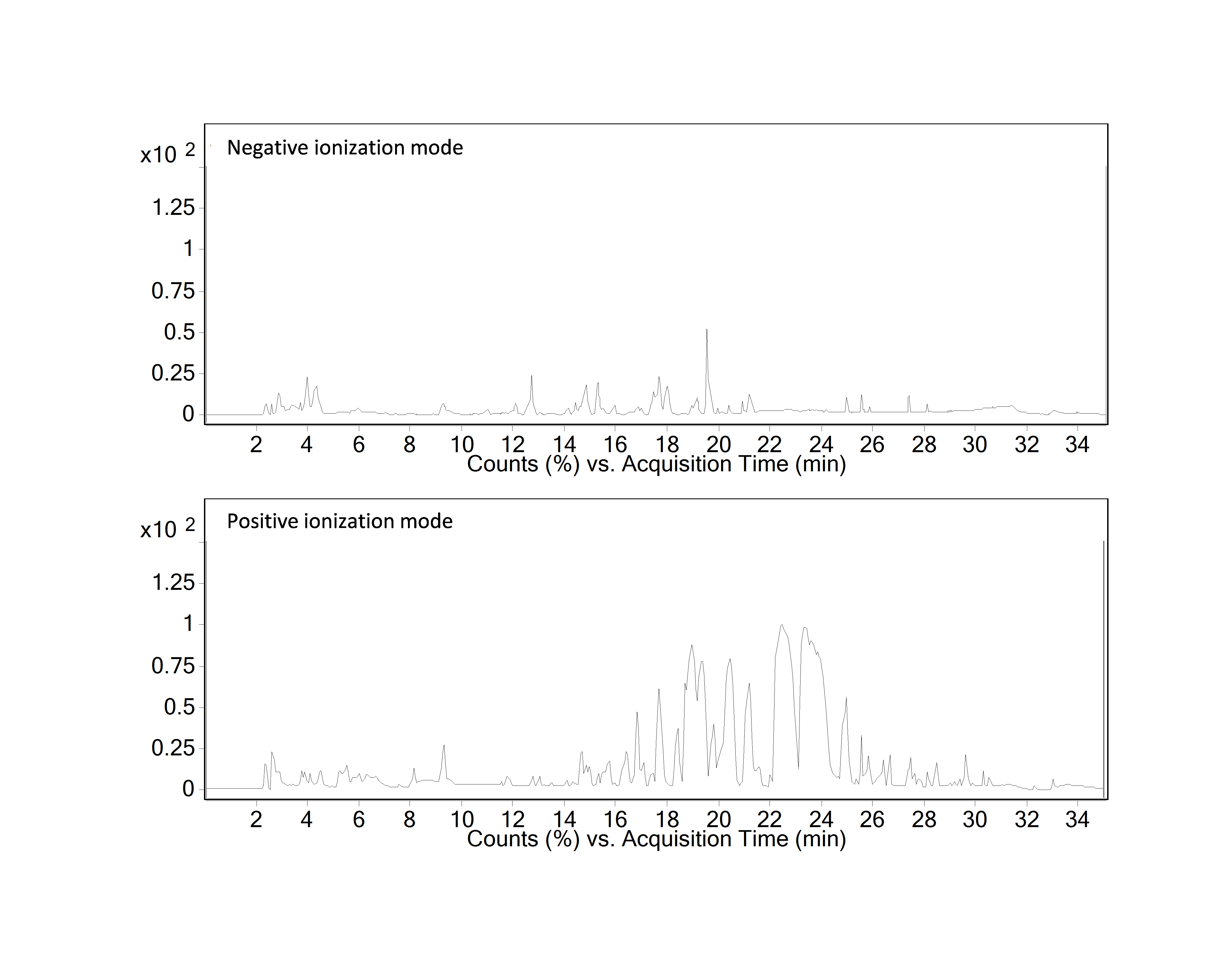

Supplement: Supplementary file 1 — Figure S1: Base peak chromatogram of F. capreolata extract determined by RP-UHPLC-DAD-QTOF-MS and elecrospray ionization in the negative and positive ionization modes. Table S1: Characterization of alkaloids from F. capreolata extract by GC-QTOF-MS. Table S2: Characterization of alkaloids from F. capreolata extract by RP-UHPLC-QTOF-MS. Table S3: Precision and accuracy of the method proposed. [file 5178729.f1.tif]
