# Supplementary material for: Alkaloids Profiling of Fumaria capreolata by Analytical Platforms Based on the Hyphenation of Gas Chromatography and Liquid Chromatography with Quadrupole-Time-of-Flight Mass Spectrometry
Source: Int J Anal Chem. 2017 Nov 28;2017:5178729. doi: 10.1155/2017/5178729 (PMC5733964; doi:10.1155/2017/5178729)
Supplement: Supplementary file 4 [file 5178729.f4.docx]

**Table S3.** Precision and accuracy of the method proposed.

|  | Intraday | | Interday | |
| --- | --- | --- | --- | --- |
| Concentration (nanomol/mL) | Repeatibility, RSD^a^ (%) | Accuracy (%) | Repeatibility, RSD^a^ (%) | Accuracy (%) |
| 256 | 0.5 | 100 | 2.2 | 99 |
| 64 | 4.2 | 95 | 7.4 | 95 |
| 8 | 3.1 | 106 | 7.9 | 110 |

^a^RSD, residual standard deviation.
